# Comparison of Online Resilience and Psychological Safety Courses for Canadian Public Safety Personnel

**DOI:** 10.3390/ijerph23050564

**Published:** 2026-04-27

**Authors:** Michelle C. E. McCarron, Sandra Jasinoski, Marilyn Cox, Yan Song, Joy C. MacDermid, Gregory S. Anderson

**Affiliations:** 1 Faculty of Science, Thompson Rivers University, Kamloops, BC V2C 0C8, Canada; michelle.mccarron@uregina.ca (M.C.E.M.); ysong@tru.ca (Y.S.); 2 Department of Psychology, University of Regina, Regina, SK S4S 0A2, Canada; 3 School of Rehabilitation Therapy, Queens University, Kingston, ON K7L 3N6, Canada; m.cox@queensu.ca; 4 School of Physical Therapy, Western University, London, ON N6A 3K7, Canada

**Keywords:** resilience, online learning, public safety personnel, first responders, resiliency training, skill degradation, mental health literacy, operational stress, psychological safety, public safety families

## Abstract

**Highlights:**

**Public health relevance—How does this work relate to a public health issue?**

- Public safety personnel have high rates of posttraumatic stress injury.
- Mitigating the harmful impacts of trauma exposure is a worthy endeavor.

**Public health significance—Why is this work of significance to public health?**

- Leaders in public safety are often left to make decisions about implementation of mitigation strategies (psychoeducation) without a clear understanding of all available options, evidence supporting these options, or their content.
- In order to help public safety leaders in Canada make informed decisions about potential health promoting activities, a standard metric was applied to a variety of course options.

**Public health implications—What are the key implications or messages for practitioners, policy makers and/or researchers in public health?**

- Resilience is more than an individual trait, involving interactions between the individual, their workplace, and their family.
- Mitigation strategies should match the specific needs of the organization, and be chosen after understanding the evidence supporting each option.

**Abstract:**

Public Safety Personnel (PSP) face numerous potentially psychologically traumatic events in the line of duty. Resilience courses intended to mitigate the effects of operational stress injuries in this population—many of which are available online—have proliferated in recent years. An environmental scan yielded 15 courses that met all inclusion criteria. Courses were required to be offered for $250 or less and had to be created and/or hosted in Canada. Courses focused on individual (*n* = 7), family (*n* = 2), or workplace (*n* = 6) resilience. A qualitative content analysis was conducted. Data were extracted from public-facing documents and course materials, and supplemented by additional text materials and contacting program staff for clarification, when necessary. Coding and synthesis were completed in iterative team meetings. Courses were compared across numerous dimensions, including focus, intended audience, cost, enrollment details, length, instructional style, customization for PSP, and completion requirements. Nearly half of the courses (46.7%) were available free of charge. Outcome data were available for four of the courses, with most studies showing initial gains in resilience, skills, knowledge, attitudes, intentions, and/or behaviors, but with evidence of skill decay over time. The potential benefit of short refresher sessions warrants further investigation.

## 1. Introduction

Public Safety Personnel (PSP) comprise an array of emergency response professions (e.g., police, fire, emergency medical services, public safety communications, search and rescue) and other services dedicated to protecting the public (e.g., corrections, border security, intelligence services). By virtue of their roles, PSP are at an increased risk of exposure to potentially psychologically traumatic events (PPTEs) [1]. Repeated exposure to PPTEs can contribute to the development of post-traumatic stress injuries (PTSIs), which extend beyond posttraumatic stress disorder (PTSD) to include a wide range of mental health issues such as depression and anxiety [2]. The impact on the individual PSP can be devastating, affecting their overall well-being, relationships, and ability to perform their duties effectively. From an organizational perspective, the high prevalence of PTSIs and other operational stress injuries (OSIs) among PSP can result in increased job attrition, absenteeism, and reduced productivity [3,4,5]. This places a substantial burden on PSP organizations, which must allocate resources to recruit, train, and support replacement personnel, as well as manage the disruptions caused by mental health-related absences.

The societal costs of PTSIs and other OSIs among PSP are also substantial. The increased suicide rates observed in this population represent a tragic loss of life and have far-reaching consequences for families, communities, and the broader public [6]. Moreover, the mental health challenges faced by PSP can compromise their ability to fulfill their essential duties of protecting the public, potentially jeopardizing public safety and security. In response to a 2016 report by the Standing Committee on Public Safety and National Security identifying the urgent need for a national strategy to address the mental health needs of PSP, the Canadian Institute for Public Safety Research and Treatment (CIPSRT) was established as a result of dedicated funding announced in the 2018 federal budget [7].

Research into the prevalence, contributors, and mitigators of PTSIs and other OSIs has proliferated in Canada within the last decade. The Canadian Institutes of Health Research (CIHR) has awarded more than $12 million in funding since 2019 through funding programs dedicated to PSP mental health research, in addition to numerous PSP mental health-related projects supported via other funding calls [8]. Recently, attention has also turned to the impacts of PPTEs and OSIs experienced by PSP on their partners, children, and other family members. The COVID-19 pandemic increased the mental health challenges faced by PSP, who were at heightened risk of exposure to the virus while fulfilling essential services [9]. Particularly in the earlier days of the pandemic, when little was known about transmissibility, worldwide death tolls were on the rise, and a vaccine was not yet available, existing risks were further amplified by the stress and worries about the potential implications that exposure to the virus could have on them personally, as well as those with whom they cohabitated.

### 1.1. Online Resiliency Training

One promising approach to supporting the mental health of PSP is the development of online resilience and psychological safety courses. This delivery format offers several benefits, including increased flexibility and accessibility, particularly for those in rural, remote, and First Nations communities [10]. Online courses can accommodate the unique scheduling demands of PSP, who often work shift-based schedules, by providing self-paced options that can be accessed at any time. Moreover, the online delivery of these courses can improve accessibility for individuals in less populous areas, provided that sufficient telecommunications infrastructure is in place. By offering resilience and psychological safety training in an online format, PSP can access evidence-based support and resources that can help mitigate the significant personal, organizational, and societal costs associated with OSIs. As the mental health landscape continues to evolve, it is crucial that PSP, their leaders, and mental health professionals have access to the most current and relevant information to guide their decision making. However, it cannot be presumed that online delivery can solve access issues while providing similar efficacy to in-person delivery. Firefighters acknowledge the convenience and cost-effectiveness advantages of online delivery, while also reporting that they may be less engaged during online training [10].

Pandemic-related restrictions in travel and social gatherings, concerns about minimizing unnecessary risk of exposure, and the unique occupational stressors that the pandemic created for PSP [11] further compelled numerous practitioners and researchers in the field of PSP mental health to adapt existing face-to-face resiliency programming into online modalities and/or to create new opportunities for resiliency-based learning. In response the burgeoning need to support the mental health of those working on the front lines during the pandemic, the Public Health Agency of Canada (PHAC) funded 13 projects via a $50 million commitment in the federal government’s Budget 2021 to address the mental health impacts of the pandemic on PSP, frontline healthcare workers, educators, and families of essential workers [12]. Among these projects were four that involved expanding or adapting existing individual resilience programs (Before Operational Stress, Resilient Minds, Road to Mental Readiness, and The Working Mind), and creation of a resilience program focused on family members of PSP (PSPNET Families) [13].

### 1.2. History of Resilience Courses for PSP in Canada

Although research on resilience dates back 50 years within the realm of childhood development [14,15], research into the role of resilience in supporting the mental health of PSP is a more recent development. Resilience is a dynamic process of adapting and recovering in the face of challenges and is associated with a constellation of protective personality characteristics such as self-efficacy, self-esteem, hopefulness, positive affect, and emotional self-regulation [14,16]. While early research focused primarily on individual resilience factors, focus has since expanded to include resilience at the family, community, and cultural levels [15,16]. Research into resilience among military personnel and PSP saw an uptick in the early 2000s in the wake of global combat in the Middle East and the 2001 terror attack in the United States (U.S.). Adopting a positive psychology framework, the U.S. military launched its Comprehensive Soldier Fitness (CSF) program in 2008 [17], which included individual assessment, a resilience training program, and a Master Trainer program to aid in disseminating training throughout the U.S. military.

Concurrently, similar efforts to create a resiliency training program were underway in Canada. A self-paced online resiliency training program was developed and delivered first in 2011 by the Justice Institute of British Columbia (JIBC) to help paramedic students in training prepare for their first experience riding along with a preceptor in an ambulance [18]. Using the same course, improved resilience in Bachelor of Science in Nursing students was found at both 1 and 3 months post training [19]. Despite research in paramedic and nursing settings demonstrating improved resilience and reduced negative mental health outcomes among trainees, skill decay was noted, with improvement noted in resilience scores at 3 months but not at 6 months post training [20]. Results suggest that developing skills to mitigate and manage workplace trauma can reduce or help mitigate the negative impact of exposure to trauma and potentially reduce the risk of developing trauma-related mental health problems in both paramedic and nursing trainees.

During this time period, the Canadian Department of National Defence (DND) developed the Road to Mental Readiness (R2MR) program [21]. In 2018, this training was generalized for a broader PSP audience by the Mental Health Commission of Canada (MHCC) and was rebranded as The Working Mind for First Responders (TWMFR), with The Working Mind (TWM) being adapted for a general audience. Since that time, the program has been tailored for other audiences, including those in the legal sector, sports, healthcare workers, long-term care workers, and tradespersons [22]. The original R2MR course was adapted for delivery via an online learning platform and video conferencing; this new version—ER2MR—was delivered by CIPSRT from 2022–2024 as part of a larger training program funded through the PHAC initiative to address PTSD and trauma among frontline workers in the wake of the COVID-19 pandemic [13].

Around the same time as the original launch of MHCC’s adaptation of R2MR for the Calgary Police Service, another resilience program—this one geared toward firefighters—was developed, launching in 2016 [23]. Initially intended to be a local initiative designed to meet an identified need within Vancouver Fire and Rescue Services (VFRS) in the province of British Columbia (BC), Resilient Minds expanded nationally in 2020 [23]. Since that time, Resilient Minds has been further tailored, with adaptations offered for volunteer firefighters, wildland firefighters, emergency communications operators, and Indigenous first responders [24]. Similar to R2MR and TWM, Resilient Minds has been adapted for virtual delivery [25]. As with ER2MR, this project received funding through PHAC’s pandemic-related funding initiative for frontline first responders for adaptation, pilot testing, and evaluation of the Indigenous firefighters course, as well as a French translation of Resilient Minds [13].

One year following the launch of Resilient Minds’ initial course with VFRS, Wounded Warriors Canada (WWC) released its Trauma Resiliency Program (TRP) with the aim of aiding serving military members, Veterans, and first responders in developing resiliency skills to address OSIs [26]. The Before Operational Stress (BOS) training program was introduced the following year, also in collaboration with WWC [26]. Initially offered as in-person group-based sessions, BOS was adapted, pilot tested, and evaluated for virtual delivery in an asynchronous format as part of the PHAC funding initiative during the pandemic [13]. The course has since been tailored to other specialized audiences, with versions of the online course offered for PSP, military members, healthcare services, and legal services. An online version of the synchronous, facilitated group course is also offered.

In 2015–2016, the Building Resilience course was first developed by JIBC personnel [18]. The Three Pillars of Resilience Program for Public Safety Personnel conceptualizes resilience as an ecosystem comprising personal, family, and workplace factors, and recognizes that events in any of the three categories can impact PSP resilience [27]. As such, individual, family, and workplace resilience courses were developed for public safety employees (PSE, which includes PSP and civilian workers) with the goal of improving their resilience through increased knowledge and awareness of personal, family, and workplace factors impacting their psychological health and wellbeing. The impact of this training is being investigated through a pilot study of six public safety organizations (in the fire and police sectors) from the Lower Mainland of BC.

As was the case with ER2MR, Resilient Minds, and BOS, an additional resource called PSPNET Families was also funded through PHAC’s pandemic initiative [13]. Building upon an existing collection of therapist-guided and self-guided internet-based cognitive behavioral therapy (ICBT) courses for Canadian PSP, this funding supported development of a self-guided Spouse or Significant Other (SSO) Wellbeing Course, as well as a comprehensive set of resources including information, strategies, and skill-building exercises.

### 1.3. Purpose and Objectives

The main purpose of the present study is to provide a comprehensive structural overview of the many options available to Canadian PSP and their families for virtually delivered resilience courses. This research has been undertaken in response to questions that have arisen within the Canadian PSP community regarding how to determine—in light of the abundance of courses now available—the degree to which these courses are backed by evidence and have been customized for PSP and their families. Despite the growing number of resilience training programs available to Canadian PSP, there is limited comparative information regarding their structure, delivery methods, and evidence base. The specific objectives of this review extend beyond a simple comparative overview, however. By providing comparisons of these courses based on a range of criteria (e.g., length, instructional style, cost, completion requirements), the authors aim to assist readers in selecting the programs that best meet the unique needs of their organizations and personnel, and to equip PSP, their leaders, and mental health professionals with the necessary information to navigate the evolving landscape of online resilience and psychological safety courses. The secondary objective is to provide an overview of the evidence pertaining to program effectiveness, for those programs where such research has been published. Although these courses are specific to the Canadian context, information about factors that may be considered when evaluating resilience courses in general, as well as a review of evidence about course effectiveness, are expected to be of interest to a broader readership.

## 2. Materials and Methods

An environmental scan was conducted to identify online resilience courses for PSP, using a combination of public databases about available resilience courses, online keyword searches, and key informant recommendations. A purposive sampling approach was used, drawing from multiple sources to compile a comprehensive list of course offerings. Research Ethics Board approval was not required, as the study did not include human participants or their data.

### 2.1. Course Identification and Selection

To be included, courses had to address resilience skill development, have a psychoeducation focus, be geared or marketed toward PSP (including families), and be offered online (synchronously or asynchronously). The team agreed via consensus to exclude courses for which the cost exceeded $250 CAD, in order to keep the focus on courses that would not be prohibitively expensive to individual PSP. Additionally, courses were excluded if the focus was on treatment rather than prevention. Finally, courses were excluded if they were created/hosted outside of Canada, so as to focus specifically on content and contexts relevant to PSP in Canada. A literature search was then conducted for all of the courses. Articles reviewed in this manuscript were included if data were collected at two or more time points.

**Figure 1:**
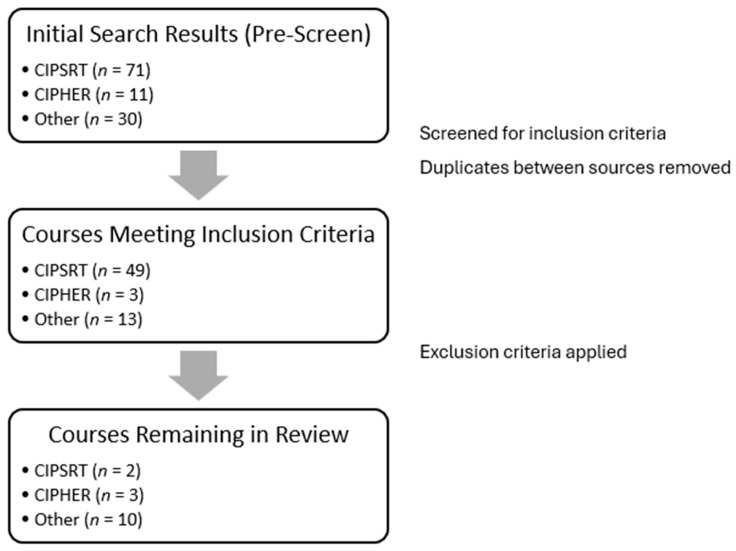
Course identification and selection process.

The initial source for information about available courses was an existing online compendium of PSP mental health training created by CIPSRT (https://www.pspmentalhealth.ca/). Services in the online database were filtered to select those that were available for individual registration and targeted toward one or more PSP groups and/or their families, with “resiliency” as a mental health focus and either “online (asynchronous)” or “online (synchronous)” as the delivery mode. This yielded 73 initial matches, with two removed as duplicates and 22 being screened out immediately for not meeting the inclusion criteria (Figure 1). Of the 49 remaining courses, two met the criteria to be included in this review.

As the CIPSRT resource was last updated in 2023, the second major source of information was the website of the Canadian Institute for Pandemic Health Education and Response (CIPHER) [28], which served as a knowledge hub for the other projects funded under PHAC’s initiative to address PTSD and trauma among frontline workers most affected by the COVID-19 pandemic [29]. Of the 11 projects listed, four were eligible for inclusion, with one being a duplicate of a course listed in the CIPSRT resource. None met the criteria for exclusion. An additional 11 potential courses were identified through a combination of the authors’ existing knowledge (all of whom have worked within the PSP mental health resilience realm in various capacities), inquiries with colleagues in the PSP mental health field in Canada, and online keyword searches (e.g., “online resilience training,” “mental health resiliency course,” “PSP mental health course”).

Although not a resiliency course as such given that the focus is on identifying potential mental health needs in others and providing support, an exception was made to include Mental Health First Aid (MHFA) in this review. The rationale for this decision was that, as one of the earliest mental health-related courses available to PSP in Canada, its popularity has been well-established, and versions specifically tailored for police and Veterans have been offered. This has made MHFA a popular choice among PSP audiences, with the intended purposes and outcomes of MHFA versus resiliency-focused courses being conflated at times. As an example, in the pspmentalhealth.ca database, the mental health focus for MHFA for Police was listed as “resiliency.” Thus, for practical reasons, the decision was made to include the course as part of this review.

An additional resource (PSPNET Families), although not a “course” per se, was included as it is open access and addresses resiliency-related topics for PSP families (however, for the purpose of this review, it is referred to as a “course,” for clarity). PSPNET Families, at the time of review, included 23 individual information pages and 21 pages of strategies and/or skill-building activities. Site content was organized into “learning,” (information pages), “trying” (strategies, skill-building, and other activities for families and couples), and “being,” which directs users to the PSPNET SSO Wellbeing course. The PSPNET SSO course itself was not included in this review, as it is structured as an ICBT course and mental health intervention courses were excluded from this study.

Two other courses identified by the authors were excluded due to being directed toward mental health care practitioners working with PSP, leaving 10 studies from the third list and bringing the total number of courses reviewed in this study to 15.

### 2.2. Procedure

The researchers used a multi-pronged approach to gather information about the online resilience courses for PSP. The primary source of data was the programs’ websites, from which the researchers collected information on various aspects of the courses. This included program history, such as the origins, contributors, and funding. They also gathered contact details such as the primary contacts and marketing information, when available. The researchers documented the courses’ goals, which ranged from skill development to raising mental health awareness. They noted details about enrollment, including the cost and any limits or confidentiality policies. Data were extracted and iteratively reviewed by the project team, to assess completeness, accuracy concerns, and potential for obtaining more detail where needed.

One or more members of the project team enrolled in the courses, where possible, to corroborate any information about the courses that was gleaned from public websites and to ensure that information provided in the comparison matrix is complete and accurate. Of the 15 courses reviewed, eight are normally restricted to PSP only. In four cases (i.e., ER2MR, TRU-I, TRU-W, TRU-F), the co-authors of this paper were involved in course development and thus were permitted to enroll. For one course, participation in the course itself was not possible due to its restriction to firefighters; however, a team member (J.C.M.) with prior research experience, as well as the original course creators, were able to confirm the details. One asynchronous, self-directed course did not require special permission to enroll (BOS), and for two courses, the standard version was taken instead (TWM and MHFA). To mitigate potential bias, coding for each course was reviewed by multiple team members to ensure that content pertaining to courses did not reflect the perspectives of only those who were connected to the course in some way. Additionally, some of the courses created, co-created, or evaluated by the authors could be seen to “compete” with one another (e.g., ER2MR vs. RM vs. the TRU-I course). None of the authors stood to benefit financially from the findings: ER2MR and the TRU courses were/are offered free of charge and the co-author whose research has included RM (J.C.M.) has had no involvement in course development or delivery. The consensus approach taken by the authors nevertheless ensured that the viewpoints of multiple team members—including those who did not have any actual, potential, or perceived conflicts of interest—were represented.

Following course completion, information about lesson structure (i.e., number of lessons, objectives provided, slides, videos, case studies, end-of-unit summaries, workbook/activity sheets, interactive features, handouts/reading material), learning assessments (i.e., quiz requirements, explanations for incorrect options, ability to retake quizzes, ability to build on previous attempts), and course completion requirements (i.e., proof of completion, eligibility for continuing education credits), were verified and expanded upon. While some program sites provided links to published evaluations and research about their courses, this information was primarily compiled via literature searches conducted using PubMed, PsycINFO, and Google Scholar.

### 2.3. Analysis

The authors used a qualitative content analysis approach to examine the information pertaining to the online resilience and psychological safety courses that met the inclusion criteria. A mixed deductive/inductive approach was used. First, a comparison matrix consisting of pre-determined variables was created by four members of the research team, using a consensus approach, prior to reviewing the courses. Additionally, an inductive approach was employed as courses were coded, in order to identify similarities and differences beyond the pre-specified variables, again using a consensus approach to decision making. This enabled the research team to gain a comprehensive understanding of the courses, including their underlying theoretical perspectives, pedagogical models, and specific skill-building components. The iterative nature of the content analysis framework involved the team reviewing the data, identifying any discrepancies, and resolving them through consensus. This collaborative process ensured the accuracy and reliability of the information presented in the comparison matrix.

## 3. Results

Brief overviews of all courses (Table 1; Appendix A) and key features (Table 2) are provided. ER2MR and the three TRU courses are not currently being offered, as of this writing, although the pilot-tested TRU courses are expected to become available to all in 2026. ER2MR course material was only available for a few days after the course ended by logging into the learning management system. Core learning content could not be downloaded for future reference. Handouts, activities, and workbooks, as applicable, were available for offline use. As post-completion access periods varied widely across courses, course participants are generally limited in what material they may refer back to at a later date.

### 3.1. Course Features

Courses fell into three broad categories: individual (*n* = 7), workplace (*n* = 6), or family (*n* = 2). Eight courses were geared toward PSP audiences (Table 1). Of these, five were developed specifically for PSP. In some cases, the same provider offered multiple courses that were included: TRU (i.e., TRU-I, TRU-W, TRU-F), CCOHS (i.e., PHSW, PHSE, RMHSW), and MHCC/Opening Minds (i.e., TWM, BTS). All courses were available in English, with 10 also being available in French (Table 2).

Enrollment fees ranged from $0–255, with seven courses being free of charge (Table 2). Two courses (TWM) had variable pricing, as set by the individual facilitators. For TWM, current offerings as of this writing were priced to a maximum of $255; however, other sessions fell within the price range for the study inclusion criteria. An additional course (RM) was offered free of charge to Canadian Association of Fire Chiefs (CAFC) members. Most courses that were offered free of charge were funded or subsidized through funding from CIHR, PHAC, or mental health grants from industry donors. Seven courses (i.e., TWM, RM, MHFA, PHSW, PHSE, RMHSW, BTS) were offered by Canadian mental health and occupational health organizations, five courses (i.e., ER2MR, PSP-F, TRU-I, TRU-F, TRU-W) were created at Canadian universities, and three courses (i.e., BOS, CBS, EPHWR) were offered by private/for-profit groups. ER2MR is not being offered as of this writing. The three TRU courses are not currently offered as the pilot study has ended, but they are anticipated to be updated and released free of charge in 2026.

Eleven courses were fully online, self-directed, asynchronous courses. BOS also offers facilitated options (virtual or in-person delivery) in addition to its asynchronous “on demand” course. ER2MR was offered in a blended format. The majority of learning hours in ER2MR were dedicated to facilitated instruction; however, asynchronous preparation and homework activities were interspersed. Three courses (TWM, RM, MHFA) are synchronous, facilitated courses with options for virtual delivery, with a small amount of asynchronous preparatory work for TWM. All facilitated courses involved group participation as a component. Courses ranged from 30 min in length (RMHSW) to up to 25 h (ER2MR). One course (TWM) offers three brief “refresher” modules three months post-course, which can be completed asynchronously.

Courses included multi-modal content, with various combinations of text, slides, videos, case studies, interactive features (e.g., polls, activities), and handouts/workbooks (Table 3). Courses offering certificates of completion required completion of an end-of-course quiz or end-of-module quizzes for self-directed courses, and attendance for the entire course but no quizzes for facilitated courses. One course provided information about eligibility for continuing education credits (EPHWR).

### 3.2. Course Effectiveness

For four courses, published research was available in which the effectiveness of the courses in improving resilience, skills, knowledge, attitudes, intentions, behaviors, and/or mental health was measured post course and at one or more follow-up periods (Table 4). Studies were included if, at a minimum, pre- and post-course data were collected. Descriptive information about these studies, including participants, methodology, and a summary of findings, is also provided (Table 5). As the Canadian MHFA was a direct offshoot of a course offered internationally, the volume of published research was prohibitive; instead, for MHFA, meta-analyses were included and any studies identified via the literature search were removed if they were already addressed in one or more meta-analyses. One meta-analysis was excluded from Table 4 and Table 5 because the authors of the meta-analysis found a high risk of bias in the studies identified and there was a lack of outcomes at their specified timepoints of six months and one year post course completion [30].

Additional research describing course satisfaction or data collection at a single timepoint was available for RM, ER2MR, and PSPNET Families, but is not included in this table, as potential improvements in these domains could not be ascertained. Follow-ups ranged from 16 days to 12 months. Most courses did not lead to observable improvements in mental health; in the few instances where statistically significant improvements were observed, effect sizes were small and these were either not sustained long term or were complicated by high within-subject variance. Improvements with small to moderate effects sizes were observed for the other variables of interest in several studies; however, gains were commonly observed to deteriorate over time.

## 4. Discussion

The results of this review demonstrate the diversity of online resilience and psychological safety courses available to Canadian PSP, reflecting the considerable investment in improving PSP mental health that has been made in Canada over the past decade. This historical context has been provided in addition to the overview and comparison of the resilience courses themselves, in order to contextualize why and how such courses have proliferated in Canada in recent years. This information may be of interest to PSP, mental health professionals, and policy makers in other countries where similar efforts are underway or are being considered.

The courses included in this analysis employ a range of modalities, from self-directed, asynchronous learning to facilitated, synchronous sessions with video conferencing. This variety in delivery styles allows PSP to access resilience training that best fits their individual needs and preferences, whether they require the flexibility of self-paced learning or the guidance and support of a live instructor. Similarly, the courses vary in depth and detail of content covered. Some offer a more comprehensive, multi-week program, while others provide a focused, single-session intervention. This range enables PSP to choose courses that align with their specific learning objectives, whether they are seeking a broad introduction to resilience-building strategies or a more in-depth exploration of a particular topic.

The differences in the theoretical frameworks and pedagogical models underlying the courses have important implications for their effectiveness and suitability for PSP. Courses that adopt a more learner-centered approach, with a focus on skill development and practical application, may be better suited to the needs of PSP, who often require tangible, job-relevant strategies to manage the unique stressors they face. In contrast, courses with a more facilitator-centered, didactic style may be less engaging and less effective in fostering the active engagement and self-reflection necessary for resilience building. However, empirical evidence is yet to be provided comparing different teaching modalities for the same content in this area.

Additionally, the variability in the available evidence supporting the courses is noteworthy. While some programs have been rigorously evaluated with peer-reviewed studies demonstrating their efficacy, others rely on more limited, internal program evaluations. This disparity in the strength of evidence base may impact the confidence with which PSP, leaders, and mental health professionals can recommend and implement these courses.

An important consideration is the potential benefits of the courses designed specifically for PSP families. Given the growing recognition of the impact of PSP work-related trauma on their loved ones [41], these family-focused programs may play a crucial role in supporting the overall well-being of PSP and the family unit. By equipping family members with resilience-building strategies and fostering a better understanding of the unique challenges faced by PSP, these courses can help strengthen the social support networks that are essential for mitigating the negative mental health consequences of these demanding professions.

Despite initial gains across domains in several published studies, even in the short-term, deterioration in resiliency scores, resiliency skills, knowledge, attitudes (including stigma), mental health-seeking intentions, and mental health-supporting behaviors deteriorated—often significantly—compared to scores obtained immediately post-course. In particular, those studies with longer follow-up periods (9+ months) commonly found declines to the extent that improvements were no longer statistically significant over baseline. These findings are consistent with other research that has noted skill deterioration over time [41]. This suggests a need for booster sessions in order to reinforce and sustain improvements. Given the considerable variation in length/hours for completion among the courses reviewed, it may be the case that longer courses may be beneficial for initial psychoeducation, with shorter courses potentially serving as booster sessions. One of the courses reviewed, TWM, offers three brief self-directed, asynchronous booster modules, which become available to course participants three months following course completion [22].

### 4.1. Limitations

A significant limitation of this review is the challenge of maintaining up-to-date information on the rapidly evolving landscape of online resilience and psychological safety courses for Canadian PSP. As new programs are developed and existing ones are updated or expanded, the details provided in this review may become outdated. For instance, St. John’s Ambulance Canada, which is one of the organizations offering MHFA in Canada, launched a new virtual Emotional First Aid Training program in Fall 2025 (after the course reviews for this study were completed), with frontline workers as one of their focuses [42]. Further, during the pandemic and, at times, for local contextual reasons, programs have been adapted, meaning that fidelity is not only problematic but often not reported. To ensure that PSP, leaders, and mental health professionals have access to the most current information, the authors recommend the creation and ongoing maintenance of an online database that catalogues these courses, with periodic reviews and updates.

Another limitation is the restricted accessibility and availability of some courses. The authors note that some courses were restricted to PSP only, limiting the ability of the research team to directly evaluate those programs. Additionally, the team found an accessibility checker was ineffective due to the variability in the presentation of the online content, which prevented a comprehensive assessment of compatibility with assistive technologies. It is also important to acknowledge the limitations in the availability of course registrations, particularly for those programs that require a live facilitator or group participation, which may pose challenges for PSP who are interested in accessing these more interactive learning modalities.

Additionally, it should be noted that the inclusion criteria focused on Canadian courses specifically, as the intent is to provide a comparison of courses that are primarily marketed to Canadian PSP audiences. Despite this focus, the overall findings are expected to be of relevance to a broader audience. Courses were also limited to those that were $250 or less, in order to focus on training that would not require a substantial financial investment for PSP and their families. However, it should be noted that of the seven courses identified in the initial search that were excluded solely on the basis of cost, all were offered by the same service provider and there was no published evidence about their effectiveness.

Finally, the authors acknowledge that the main purpose of this review was not to evaluate the effectiveness of the online resilience and psychological safety courses for PSP. Information has been provided about the structural aspects of the programs, not their effectiveness for protecting or improving PSP mental health and resilience. This information was augmented by the inclusion of a section summarizing literature regarding the effectiveness of programs in improving resilience where such pre- vs. post-course comparisons had been conducted. Nevertheless, it is noteworthy that no randomized trials compared different programs, so effectiveness evidence is limited [20]. The process for co-designing and testing complex interventions has been described and is underutilized in the programs we evaluated [43,44].

Rigorous program assessments are a significant limitation, as the authors recognize the importance of understanding the tangible benefits of these interventions for the PSP community. Notably, many of the courses reviewed have not been rigorously evaluated, highlighting the need for future research to assess the effectiveness of these online programs. In a systematic review and meta-analysis examining proactive psychological programs designed to mitigate PTSIs, Di Nota et al. [45] found “modest evidence for time-limited reductions in PTSI following participation in holistic programs that promote resilience, stress, and emotion regulation among at-risk workers” (p. 2). Importantly, using a structured quality assessment of the 42 papers reviewed, 30 were rated as low and medium to low quality, and only one high, while 14 were identified as having a high risk of bias. Conducting more high quality, longitudinal randomized controlled trials, with longer follow-up periods and larger sample sizes to account for attrition, would bolster the evidence for course effectiveness. Evaluating how these courses improve mental health outcomes, resilience, and other relevant factors, as well as considerations such as cost effectiveness and comparisons of the effectiveness of different pedagogical approaches, is crucial to ensure PSP have access to the most impactful resources. The authors suggest that future research should focus on assessing the measurable benefits of these online interventions for the target population.

### 4.2. Future Directions

To ensure that PSP and their organizations have access to the most relevant and impactful online resilience and psychological safety courses, the authors recommend several key next steps. First, the development of a gap analysis or needs assessment tool would be a valuable addition to this work. By providing a structured framework for PSP leaders and organizations to identify the priority topics and skills for their personnel, such a tool could help guide the selection and implementation of the most appropriate online courses. This could involve modifying existing frameworks, such as the British Columbia First Responders’ Mental Health (BCFRMH) gap analysis [46], to specifically address the unique needs and challenges faced by the PSP community.

Additionally, the authors suggest expanding the scope of this review to include online resilience courses from other countries. While the current analysis focused on Canadian offerings, the inclusion of international programs, including those where English is not the majority language, may provide additional insights and perspectives that could benefit broader PSP populations. Comparing the theoretical foundations, pedagogical approaches, and evidence base of courses from multiple countries could yield valuable information to guide future program development and implementation.

The authors further recommend a critical evaluation of the theoretical frameworks underlying the different online resilience and psychological safety courses. Given the variability in the theoretical models and pedagogical approaches observed in the current review, a deeper examination of the relevance and utility of these foundations could inform the selection of the most effective courses for PSP. This analysis should consider the alignment between the theoretical underpinnings of the courses and the unique needs and experiences of PSP, whether different approaches are better suited toward particular objectives, and the evolving evidence base in the field of trauma-informed, resilience-focused interventions.

In the course of reviewing the learning content, it was also noted that the extent to which in-text citations and references were provided, as well as the perceived quality of those citations, varied considerably. A more in-depth content analysis using a quality appraisal framework would be beneficial; however, this is complicated by copyright restrictions unless such use of materials is deemed to fall under fair use. Regardless, statements to which participants must agree prior to accessing the material for learning purposes may nevertheless preclude such use without express permission. Some programs, particularly those who offer their courses for profit, may be disinclined to agree to such a review. Nevertheless, such a review would provide important information about both the quality and current relevance of the research on which the topics addressed in such courses are based.

Ultimately, decisions on content and delivery can affect program outcomes. Ideally, pre-trial research would establish the “active ingredients,” and rigorous randomized trials would establish effectiveness. The culture of urgent need may have ultimately contributed to the uncertainty in the evidentiary pool since the accepted methods for rigorous research have been underutilized.

By pursuing these future directions, the support available to Canadian PSP can be strengthened, ensuring that they have access to online mental health resources that are tailored to their specific needs, evidence-based, and supported by rigorous evaluation.

## 5. Conclusions

The comparison of online resilience and psychological safety courses for Canadian PSP presented in this review provides an overview of the diverse range of programs available, highlighting their varied approaches, delivery methods, and evidence base. By equipping PSP with practical, evidence-based resilience-building strategies, these online courses have the potential to mitigate negative mental health outcomes, ultimately benefiting not only individual PSP but also their organizations and the communities they serve. Furthermore, the inclusion of family-focused programs recognizes the far-reaching impact of PSP work-related trauma, underscoring the importance of supporting broader social networks.

As mental health resources continue to evolve, it is essential that PSP, their leaders, and mental health professionals have access to the most current and relevant information to guide their decision making. While numerous resilience and psychological safety programs are available to Canadian PSP, robust evidence supporting sustained long-term effectiveness remains limited. By addressing the limitations identified in this review and pursuing future directions, researchers and practitioners can help ensure that Canadian PSP have access to online resources tailored to their specific needs and that contribute to the overall wellbeing and resilience of those who dedicate their lives to protecting the public.

## Figures and Tables

**Table 1:** Overview of resiliency courses for Canadian PSP.

Course	Organization	Website
Individual		
BOS	Wayfound	https://www.beforeoperationalstress.ca/all-programs
CBS	CPKN	https://www.cpkn.ca/en/course/creating-brave-spaces/
ER2MR	CIPSRT (w/DND)	https://www.cipsrt-icrtsp.ca/en/training-opportunities
MHFA	MHCC	https://openingminds.org/training/mhfa/standard/
RM	VFRS, CMHA	https://cmha.ca/resilient-minds
TRU-I	TRU	https://openmic.tru.ca/courses/2947/
TWM	MHCC (w/DND, CPS)	https://openingminds.org/training/twm/
Workplace		
BTS	MHCC	https://openingminds.org/training/phs/building-team-success/
EPHWR	MWH	https://www.myworkplacehealth.com/
PHSE	CCOHS	https://www.ccohs.ca/products/courses/phs-employers
PHSW	CCOHS	https://www.ccohs.ca/products/courses/phs-workers
RMHSW	CCOHS	https://www.ccohs.ca/products/courses/phs-stigma
TRU-W	TRU	https://openmic.tru.ca/courses/2943/
Family		
PSP-F	CIPSRT	https://www.pspnet.ca/en/for-families-of-psp
TRU-F	TRU	https://openmic.tru.ca/courses/2945/

Note: Weblink were accessed on 31 January 2025.

**Table 2:** Cost, format, and availability of resiliency courses for Canadian PSP.

Course	Audience	Cost	French Version	Length	Schedule	Instructional Style
Individual						
BOS	Multiple versions (incl. PSP)	$210	Yes	6–8 h	On Demand: Asynchronous Classroom: Synchronous	On Demand: Self-directed Classroom (≤25): Facilitated lecture, group discussion Classroom (>25): Lecture
CBS	General	$200	No	4–5 h	Asynchronous	Self-directed
ER2MR	PSP	$0	Yes	22–25 h	Synchronous (4 days over 2 weeks) plus asynchronous homework	Facilitated lecture, group discussion, some self-directed content
MHFA	Multiple versions (incl. Police, Veterans)	$195–250 (set by instructor)	Yes	8–10 h	Synchronous (1 full day or 2 half-days)	Facilitated lecture, group discussion, some self-directed content (pre-reading)
RM	Fire	$235; $0 for CAFC member depts (2025)	Yes	8–10 h	Synchronous (2 half-days)	Facilitated lecture, group discussion
TRU-I	PSP	$0	No	4–7 h	Asynchronous	Self-directed
TWM	Multiple versions (incl. First Responders only)	$150–$255 (set by instructor)	Yes	5–7 h	Synchronous (1 day)	Facilitated lecture, group discussion, some self-directed content (pre-reading)
Workplace						
BTS	General	$69.95	Yes	2 h	Asynchronous	Self-directed
EPHWR	General	$149	No	1.5 h	Asynchronous	Self-directed
PHSE	General	$44.95	Yes	1 h	Asynchronous	Self-directed
PHSW	General	$19.95	Yes	1–1.5 h	Asynchronous	Self-directed
RMHSW	General	$0	Yes	0.5 h	Asynchronous	Self-directed
TRU-W	PSP	$0	No	3–6 h	Asynchronous	Self-directed
Family						
PSP-F	PSP, families of PSP	$0	Yes	N/A	Asynchronous	Self-directed
TRU-F	PSP, families of PSP	$0	No	3–6 h	Asynchronous	Self-directed

**Table 3:** Instructional design elements of resiliency courses for Canadian PSP.

Course	Learning Objectives	Written Material	Videos	Case Studies	Activities	Handouts/ Workbook	Quizzes	Completion Requirements	Certificate Issued
Individual									
BOS	Yes	Yes	Yes	Yes	Yes	Yes	Yes	View all pages and attempt unit quizzes	Yes (download)
CBS	Yes	Yes	Yes	Yes	Yes	Yes	No	View all pages	Yes (download)
ER2MR	Yes	Yes	Yes	Yes	Yes	Yes	No	Completion of assigned online content and full attendance	Yes (email)
MHFA	Yes	Yes	Yes	Yes	Yes	Yes	Yes	Completion of Module 1 and full attendance	Yes (email)
RM	Yes	Minimal	Yes	Yes	Yes	Yes	Optional	Full attendance	Yes (email)
TRU-I	Yes	Yes	Yes	Yes	Yes	No	No	View all pages	Yes (download)
TWM	Yes	Yes	Yes	Yes	Yes	Yes	No	Completion of Module 1 and full attendance	Yes (email)
Workplace									
BTS	Yes	Minimal	Yes	Yes	Yes	Yes	Yes	End of unit quizzes (10 questions each, ≥80%)	Yes (download)
EPHWR	Yes	Yes	Yes	Yes	Yes	Yes	Yes	Embedded knowledge check activities and final quiz (12 questions, ≥75%)	Yes (download)
PHSE	Yes	Yes	Yes	Yes	Yes	No	Yes	Final quiz (20 questions, ≥80%)	Yes (download)
PHSW	Yes	Yes	Yes	Yes	Yes	No	Yes	Final quiz (29 questions, ≥75%)	Yes (download)
RMHSW	Yes	Yes	No	No	No	No	No	None	No
TRU-W	Yes	Yes	Yes	Yes	Yes	No	No	View all pages	Yes (download)
Family									
PSP-F	Yes	Yes	Yes	Yes	Yes	Yes	No	Not applicable	No
TRU-F	Yes	Yes	Yes	Yes	Yes	Yes	No	View all pages	Yes (download)

**Table 4:** Outcomes of longitudinal assessments of resiliency course effectiveness.

Authors	Results				
	Resilience	Skills	Knowledge	Attitudes	Mental Health
Mental Health First Aid (MHFA)					
Evans et al. (2021) [31]	-	Improved Confidence with: Making contact T1–T2: *p* = 0.006 T1–T3: *p* = 0.001 Talking T1–T2: *p* = <0.001 T1–T3: *p* = 0.001 Providing help (overall) T1–T2: *p* = <0.001 T1–T3: *p* = <0.001 Providing help (depression) T1–T2: *p* = <0.001 T1–: *p* = <0.001 Deteriorated Providing help (schizophrenia) T1–T2: *p* = <0.001 T1–T3: n.s.	Improved Accurate identification of schizophrenia: T1–T2 & T1–T3, *p* <0.001 No Change Accurate identification of depression T1–T2 & T1–T3, n.s.	Improved SAQ: Schizophrenia (personal) T1–T2: n.s. T1–T3: *p* = 0.002 Deteriorated SDS: Depression T1–T2: *p* = 0.003 T1–T3: n.s. Schizophrenia T1–T2: *p* = <0.001 T1–T3: n.s. SAQ: Depression (personal) T1–T2: *p* < 0.001 T1–T3: n.s. No Change SAQ: Depression (perceived) T1–T2 & T1–T3: n.s. Schizophrenia (perceived) T1–T2 & T1–T3: n.s.	Deteriorated K-10: T1–T2: *p* = 0.012 T1–T3: n.s. No Change BMS: T1–T2 & T1–T3: n.s.
Hadlaczky et al. (2014) [32]	-	-	Included questions about beliefs re: effective treatments and ability to accurately identify a mental health problem 15 studies (*n* = 3807), mean effect size: Glass’s Δ = 0.56 (95% CI: 0.38–0.74), *p* < 0.001	Modified versions of the SDS 14 studies (*n* = 3929), mean effect size: Glass’s Δ = 0.28 (95% CI: 0.22–0.35), *p* < 0.001	-
Road to Mental Readiness (R2MR/ER2MR)					
Fikretoglu et al. (2014) [33]	-	No Change Big 4 skills quiz: n.s.	No Change Overall quiz: n.s.	No Change Self-talk summary: n.s.	-
Fikretoglu et al. (2019) [34]	No Change CD-RISC (T1,T2,T3): n.s.	-	-	-	No Change K-10, SUDS, PHQ-9, GAD-7 (T1,T2,T3): n.s.
McCarron et al. (in preparation) [35]	Improved BRS (T1–T3): γ = 0.13, *p* < 0.05; *d* = 0.251	Deteriorated Skill use (T1–T3): γ = −0.52, *p* < 0.001; *d* = 0.718 Skill helpfulness (T1–T3): (γ = −0.49, *p* < 0.001; *d* = 0.592) Confidence implementing training (T1–T3): γ = −0.52, *p* < 0.001; *d* = 0.387	Deteriorated Perceived memory for material (T1–T3): γ = −0.50, *p* < 0.001; *d* = 0.604 Resource use (T1–T3): (γ = −0.68, *p* < 0.001; *d* = 0.657)	Deteriorated Perceived resource helpfulness (T1–T3): γ = −0.52, *p* < 0.001; *d* = 0.503 No Change MHSAS (T1–T3): n.s.	Improved GAD-7 (T1–T3): γ = −0.89, *p* < 0.01; *d* = 0.269 PCL-5 (T1–T3): γ = −0.42, *p* < 0.01; *d* = 0.283 Note: High within-subject variance (SE > 5) for both No Change PHQ-9 (T1–T3): n.s.
The Working Mind (TWM)					
Carleton et al. (2018) [36]	No Change BRS (T2,T3): n.s.	-	No Change MAKS (T2,T3): n.s.	Improved OMS-WA T1: *d* = −0.287, *p* = 0.031 T2,T3: n.s. No Change UWES-9 (T1,T2,T3): n.s.	-
Dobson et al. (2019) [37]	Improved Resiliency Skills Scale T1: coefficient = −0.308, SE = 0.015, z = −20.39, *p* < 0.001 T2: coefficient = −0.186, SE = 0.026, z = −7.21, *p* < 0.001 Increased resilience: average *d* = 0.50 (range = 0.41–0.65) Deteriorated Resiliency Skills Scale T1–T2: coefficient = 0.125, SE = 0.039, z = 3.26, *p* = 0.001	-	Improved Understand how mental health problems present in workplace (T2): coefficient = −0.752, SE = 0.158, z = −8.61, *p* < 0.001	Improved OMS-WA (T1): coefficient = 0.167, SE = 0.08, z = 20.72, *p* < 0.001 Decreased stigma: average *d* = 0.38 (range = 0.15–0.51) Deteriorated OMS-WA (T1–T2): coefficient = −0.078, SE = 0.016, z = −5.02, *p* < 0.001 No Change Talk about mental health issues as freely as physical health issues (T2): n.s.	-
Dobson et al. (2021) [38]	Improved Resiliency Skills Scale Delayed (T1–T2): *t*(153) = 4.27, *p* < 0.001 Immediate (T1–T2): *t*(153) = 3.6, *p* = 0.001 Delayed (T1–T3): *d* = 0.66 Immediate (T1–T3): *d* = 0.49 No Change T2–T3 (both groups): n.s.	-	-	Improved OMS-WA Delayed (T1–T2): *t*(154) = 3.22, *p* = 0.004 Immediate (T1–T2): *t*(154) = 4.12, *p* < 0.001 Delayed (T1–T3): *d* = 0.52 Immediate (T1–T3): *d* = 0.49 No Change T2–T3 (both groups): n.s.	-
Szeto et al. (2019) [39]	Improved Resiliency Skills Scale T1: coefficient = −0.190, SE = 0.015, z = −12.90, *p* < 0.001 T2: coefficient = −0.134, SE = 0.024, z = −5.63, *p* < 0.001 Increased resilience: average *d* = 0.32 (range = 0.20–0.49) Deteriorated Resiliency Skills Scale T1–T2: coefficient = 0.076, SE = 0.022, z = 3.54, *p* < 0.001	-	Improved Understand how mental health problems present in workplace (T2): coefficient = −0.409, SE = 0.047, z = −8.61, *p* < 0.001	Improved Talk about mental health issues as freely as physical health issues (T2): coefficient = −0.195, SE = 0.037, z = −5.23, *p* < 0.001 OMS-WA (T1): coefficient = 0.123, SE = 0.008, z = 15.87, *p* < 0.001 Decreased stigma: average *d* = 0.26 (range = 0.12–0.45) No Change OMS-WA (T1–T2): n.s.	-
Thompson Rivers University Individual Resilience Course (TRU-I)					
Stoliker et al. (2022) [19]	Improved RSA T1–T2: *t*(31) = 3.03, *p* = 0.005 T1–T3: *t*(20) = 2.61, *p* = 0.017	No Change CSI-SF (T1–T2, T1–T3): n.s.	-	-	No Change GAD-7, PHQ-9, (T1–T2, T1–T3): n.s.
Vaughan et al. (2020) [20]	Improved RSA All Cohorts: T1–T2 *t*(33) = −2.769, *p* = 0.009 Cohort 2 (6 mos.): T1–T2 *t*(19) = −3.040, *p* = 0.007 Cohort 3 (9 mos.): T1–T2 *t*(5) = −3.635, *p* = 0.015 No Change Cohort 1 (3 mos.): T1–T2 *t*(7) = 1.433, *p* = 0.195	-	-	-	-

**Table 5:** Study design and summary of findings for resiliency skills courses.

Authors	Sample	Research Design	Assessments	Summary of Findings
Mental Health First Aid (MHFA)				
Evans et al. (2021) [31]	Adult family members of Veterans	Non-randomized longitudinal	T1 (baseline): *n* = 57 T2 (post-course): *n* = 57 T3 (6 months): *n* = 39	Most skills and knowledge outcomes improved at follow-up, although there was a decrease in providing help for people showing signs of schizophrenia post-course (which was no longer significant at follow-up) and no change in the accurate identification of depression. Aside from an improvement in attitudes toward people with schizophrenia by follow-up, no significant changes in attitudes were observed. Scores on a distress scale were worse following course completion, but this difference was no longer significant at 6 months.
Hadlaczky et al. (2014) [32]	International in scope, mostly adults (studies: 12 adults, 2 youth, 1 both), samples from general public non-PSP-specific groups/professions	Meta-analysis (15 studies): 9 pre/post design 4 RCTs 2 non-randomized trials with waitlist control	Follow-up periods ranged from 6 weeks to 6 months; 2 studies had no long-term follow-up	Effect sizes for measures of changes in: (a) knowledge ranged from 0.22 to 1.33, (b) attitudes ranged from −0.31 to 0.71, and (c) behaviors ranged from 0.09 to 0.72. The mean effect size within each category suggests a moderate improvement in knowledge sustained through follow-up and small improvements in attitudes and behaviors. Resilience was not assessed.
Road to Mental Readiness (R2MR/ER2MR)				
Fikretoglu et al. (2014) [33]	CAF recruits	Group RCT 4 platoons; 1 each for v.5 and v.6, with or without homework	T1 (1 day), *n* = 213 T2 (16 days), *n* = 194	There were no significant differences across time points for the overall sample, by R2MR version, or by homework/no homework.
Fikretoglu et al. (2019) [34]	CAF recruits	Group RCT Intervention Group: T1,T2, *n* = 33 platoons T3, *n* = 28 platoons Control Group: T1, *n* = 32 platoons T2, *n* = 30 platoons T3, *n* = 25 platoons	T1 (post-course) Intervention *n* = 1452 Control *n* = 1379 T2 (5 weeks) Intervention *n* = 1181 Control *n* = 1021 T3 (9 weeks) Intervention *n* = 862 Control *n* = 786	Statistically significant improvements were noted for the affective attitude and self-efficacy subscales of the CAF-MHSUQ at T1; however, effect sizes were very small. No statistically significant differences were noted for any other measures at T1 and no significant differences were found at T2 or T3.
McCarron et al. (in preparation) [35]	Police, firefighters, paramedics, public safety communicators, other PSP (ER2MR course)	Non-randomized longitudinal	T0 (baseline), *n* = 107 T1 (post-course), *n* = 51 T2 (3 months), *n* = 31 T3 (6 months), *n* = 13	Small improvements from post-course to 6 months were noted for resilience and moderate improvements were noted in help-seeking intention. Anxiety and PTSD scores exhibited small improvements over time; however, high within-subjects variance was observed for both. Skills, knowledge, and helpfulness and use of resources deteriorated over time, with small to moderate effect sizes. There were no significant changes over time for help-seeking attitudes or depression.
The Working Mind (TWM)				
Carleton et al. (2018) [36]	Municipal police taking MHCC adaptation of R2MR (later renamed TWMFR)	Non-randomized longitudinal	T0 (baseline), *n* = 147 T1 (post-course), *n* = 113 (OMS-WA only) T2 (6 months), *n* = 52 T3 (12 months), *n* = 59 Note: The highest n per item on T1–T3 in Table 1 was used; the number of matched surveys at T1-T3 was not reported.	A small improvement was observed in workplace stigma and attitudes at T1 which did not persist at T2 or T3. No other significant differences were noted. Descriptive statistics were provided for R2MR training, skill utilization, and helpfulness; however, inferential statistics were not calculated.
Dobson et al. (2019) [37]	Staff and supervisors in public (government, education, health, other) and private (energy) sectors (TWM Employee and Managers)	Non-randomized longitudinal	T0 (baseline), *n* = 1292 T1 (post-course), *n* = 1155 T2 (3 months), *n* = 415	There was significant improvement in workplace knowledge, attitude (OMS-WA only), intentions, behaviors, and resiliency from baseline to T2; however, workplace attitude and resiliency declined significantly from T1–T2. Regression analyses for two individual items on the OMS-WA (danger/unpredictability and helping behavior) were significant at *p* < 0.001 at T1; this carried through to T2 for danger/unpredictability only. Small to moderate effect sizes for improvements in resilience and stigma were reported in a separate article by Dobson et al. (2018), although it was not indicated what time points these referred to.
Dobson et al. (2021) [38]	Office staff and kitchen and maintenance staff in a provincial health authority	Cluster RCT with waitlist control 2 groups immediate intervention (office staff), 2 groups delayed intervention (kitchen and maintenance)	Baseline (T0): Delayed, *n* = 60 Pre-course (T1): Delayed, *n* = 60 Immediate, *n* = 44 Post-course (T2): Delayed, *n* = 44 Immediate, *n* = 57 2 months (T3): Delayed, *n* = 40 Immediate, *n* = 44	Given the difference in occupation types (office vs. manual labor) in the two groups, significant group effects for resiliency and MHCS limit interpretation of findings. Non-significant reduction in resiliency, attitude, and coping was observed in both groups from post-course to follow-up, but overall gains were maintained from pre-course to follow-up. No *t*-test results were reported for T1–T3 for either group, although the text indicated that all showed statistically significant improvements with moderate effect sizes.
Szeto et al. (2019) [39]	Police, firefighters, paramedics, emergency services, corrections	Non-randomized longitudinal	T0 (baseline), *n* = 5598 T1 (post-course), *n* = 4649 T2 (3 months), *n* = 854	There was significant improvement in workplace knowledge, attitude, intentions, and behaviors from baseline to T2. Resiliency was significantly improved from baseline to T2, although there was significant deterioration from T1–T2. Regression analyses for individual items on the OMS-WA were significant at *p* < 0.001 at T1 and for maintained overall gains for two items (work-related beliefs and helping behavior) at T2. Small effect sizes for improvements in resilience and stigma were reported in a separate article by Dobson et al. (2018) [40], although it was not indicated what time points these referred to.
Thompson Rivers University Individual Resilience Course (TRU-I)				
Stoliker et al. (2022) [19]	Nursing students in a BScN program	Non-randomized longitudinal	T1 (baseline): *n* = 70 T2 (1 month): *n* = 32 T3 (3 months): *n* = 21 T1 & T3 only: *n* = 15	Resilience scale scores improved from baseline to one month post-course and this improvement was maintained at 3 months post-course. No significant changes were observed for positive coping, depression, or anxiety.
Vaughan et al. (2020) [20]	Paramedic students	Longitudinal design with cohorts randomized to different follow-up lengths (3, 6, or 9 months)	T1: Pre-course baseline (all), *n* = 227 T2: Cohort 1: 3 months post: *n* = 8 Cohort 2: 6 months post, *n* = 20 Cohort 3: 9 months post *n* = 6 Follow-up for Cohorts 1–3 combined: *n* = 34	Statistically significant improvements were noted for 6- and 9-month follow-ups, but not 3 months. Generalizability is limited, as Cohorts 1 and 3 had small sample sizes at follow-up and comparability of the three cohorts at baseline to support validity of collapsing groups was not established, with a notable difference in mean resilience score between Cohort 3 at baseline (*M* = 118.66) compared to the other cohorts (*M* = 128.25, 127.95).

## Data Availability

The original contributions presented in this study are included in the article. Further inquiries can be directed to the corresponding author.

## Abbreviations

The following abbreviations are used in this manuscript:

BC	British Columbia
BCFRMH	British Columbia First Responders’ Mental Health
BMS	Burnout Measure–Short Version
BOS	Before Operational Stress
BRS	Brief Resilience Scale
BTS	Psychological Health and Safety: Building Team Success
CAD	Canadian Dollars
CAF	Canadian Armed Forces
CAF-MHSUQ	Canadian Armed Forces Mental Health Service Use Questionnaire
CBS	Creating Brave Spaces
CCOHS	Canadian Centre for Occupational Health & Safety
CD-RISC	Connor–Davidson Resilience Scale
CIHR	Canadian Institutes of Health Research
CIPHER	Canadian Institute for Pandemic Health Education and Response
CIPSRT	Canadian Institute for Public Safety Research and Treatment
CMHA	Canadian Mental Health Association
CPKN	Canadian Police Knowledge Network
CPS	Calgary Police Service
CSI-SF	Coping Strategies Inventory–Short Form
CSF	Comprehensive Soldier Fitness
DND	Department of National Defence
EPHWR	Enhancing Psychological Health, Wellness and Resilience
ER2MR	Road to Mental Readiness Online
GAD-7	Generalized Anxiety Disorder–7
ICBT	Internet-based Cognitive Behavioral Therapy
JIBC	Justice Institute of British Columbia
K-10	Kessler Psychological Distress Scale-10
MAKS	Mental Health Knowledge Schedule
MHCC	Mental Health Commission of Canada
MHCS	Mental Health Coping Scale
MHFA	Mental Health First Aid
MHSAS	Mental Health Seeking Attitudes Scale
MWH	MyWorkplaceHealth
OMS-WA	Opening Minds Scale–Workplace Attitudes
OSI	Operational Stress Injury
PCL-5	PTSD Checklist for DSM-5
PHAC	Public Health Agency of Canada
PHQ-9	Patient Health Questionnaire–9
PHSE	Psychological Health and Safety for Employers
PHSW	Psychological Health and Safety for Workers
PPTE	Potentially Psychologically Traumatic Event
PSE	Public Safety Employees
PSP	Public Safety Personnel
PSP-F	PSPNET Families
PTSD	Posttraumatic Stress Disorder
PTSI	Post-Traumatic Stress Injury
R2MR	Road to Mental Readiness
RM	Resilient Minds
RMHSW	Reducing Mental Health Stigma in the Workplace
RSA	Resilience Scale for Adults
SAQ	Stigmatic Attitudes Questionnaire
SDS	Social Distance Scale
SSO	Spouse or Significant Other
SUDS	Subjective Units of Distress Scale
TRP	Trauma Resiliency Program
TRU	Thompson Rivers University
TRU-F	TRU Family Resilience Course
TRU-I	TRU Individual Resilience Course
TRU-W	TRU Workplace Resilience Course
TWM	The Working Mind
TWMFR	The Working Mind for First Responders
UWES-9	Utrecht Work Engagement Scale–9
VFRS	Vancouver Fire and Rescue Services
WWC	Wounded Warriors Canada

## Appendix A. Overview of Resiliency Courses

### Appendix A.1. Individual Resilience Courses

#### Appendix A.1.1. Before Operational Stress (BOS)

The Before Operational Stress (BOS) program was developed to help PSP proactively address operational stress via education and skill development. The eight learning modules consist of a combination of education and exercises for practice and/or reflection. Participants begin with an introduction to stoic service culture (Module 1) and then learn about physiological responses (Module 2) and markers of operational stress (Module 3), including the arousal continuum and optimal zone of functioning. In Module 4, participants learn about automatic and distorted thoughts, cognitive interpretations, and thinking traps. Module 5 focuses on emotions, including various types of emotional responses to operational stress and how to interpret and manage difficult emotions. In Module 6, participants focus on behavior, including conditioning, avoidance, and the therapeutic role of exposure. Module 7 provides an introduction to passive, aggressive, and assertive communication styles, as well as tips for effective communication. In Module 8, participants learn about the transition model and functional disconnection and reconnection strategies.

#### Appendix A.1.2. Creating Brave Spaces

Creating Brave Spaces is a fully online, self-directed course that aims to help leaders create psychologically safe work environments by leading with authenticity and compassion, and navigating uncomfortable scenarios. In Module 1, participants are presented with a learning roadmap and an introduction to the concept of “brave spaces,” and engage in a pre-course self-assessment exercise. In Module 2, participants learn about emotional intelligence, vulnerability, and authenticity; learn to differentiate a fixed vs. growth mindset; and identify their core values. Module 3 shifts the focus to connecting with others, with emphasis on diversity and inclusion, recognizing unconscious biases, psychological safety, empathetic listening, and taking a coaching (i.e., non-directive) approach, including four phases of having a coaching conversation. Module 4 addresses reactions to conflict, conflict management styles, and approaching difficult conversations. In Module 5, participants conclude with a review of boundaries and assertiveness.

#### Appendix A.1.3. Mental Health First Aid (MHFA)

The goals of Mental Health First Aid (MHFA) are to increase mental health awareness, improve confidence to help others, reduce mental health stigma, and enhance overall mental health. The course consists of three modules. Module 1 is self-directed and is completed online, providing an introduction to the ALGES (approach, listen, give reassurance, encourage, self-care) framework and the role of a mental health first aider. Module 2 provides participants with an opportunity to practice applying this framework in various scenarios, as well as guidelines for working with people from diverse groups. In Module 3, participants learn about self-care and practice how to use the ALGES framework in crisis situations. In addition to the general course, a version tailored for Veterans is offered. A separate version that was developed for police is not currently available as of this writing.

#### Appendix A.1.4. Resilient Minds

Initially developed for PSP working in the fire sector, Resilient Minds is a four-module course delivered in a group format. Module 1 fosters awareness and understanding of psychological trauma, with a focus on recognition of signs and symptoms, the impacts of psychological trauma (including posttraumatic stress disorder, acute stress, and cumulative stress), early response, and recovery. In Module 2, participants learn about the 4R Action Toolkit—recognize, respond, refer, and reconnect—as well as about the impacts of unresolved trauma, cognitive behavior therapy (CBT), and crisis intervention. Module 3 focuses on the “trauma-informed first responder,” with an emphasis on understanding mental health and mental illness, mental health problems encountered in a first responder work environment, application of the 4R Action Toolkit, effective communication, responding to challenging and/or violent situations, and understanding resources that are available. In Module 4, the focus shifts to managing workplace stress and building resiliency, with an emphasis on risk factors, the impacts of workplace and work–life stress, recognizing warning signs, using the 4R Action Toolkit to help themselves and their colleagues, building resilience and strengthening coping mechanisms, and further exploring skills that will enhance and reinforce psychological health.

#### Appendix A.1.5. Road to Mental Readiness: Online Version (ER2MR)

The “electronic” version of the Road to Mental Readiness (ER2MR) program is an online adaptation of the R2MR course for public safety personnel (PSP), focusing on mental health awareness, destigmatization, and developing strategies for managing stress. ER2MR consists of four modules delivered in a hybrid format. Module 1 focuses on optimizing performance, providing information about stress (e.g., the differences between personal, organizational, and operational stress) and introducing the “Big Four+” approaches to controlling stress responses: goal setting, tactical breathing, progressive muscle relaxation, visualization, self-talk, and attention control. Module 2 provides a more in-depth examination of the causes of stress in PSP, recovery and coping strategies, burnout, and suicide. In Module 3, participants learn about individual zones of optimal functioning and practice using the Big Four+ strategies. Module 4 focuses on persons in crisis and teaches participants about stigma; de-escalation; suicide risk factors, warning signs, and protective factors; and negotiation training. A customized version of Module 4 for police provides additional content such as policing persons in crisis, the continuum of police response to suicide, and documentation and follow-up.

#### Appendix A.1.6. The Working Mind (TWM)

The Working Mind (TWM) consists of three modules, with the aims of: (1) improving mental health awareness and understanding, and (2) reducing mental health stigma. In Module 1, participants learn about the impact of stigma on the self, at work, and in society, as well as raising awareness of stigmatizing vs. respectful language. Modules 2 and 3 are completed during scheduled group meeting sessions. In Module 2, participants learn about the Mental Health Continuum and how to create a wellness plan. Module 3 provides participants with information about the differences between personal, organizational, and operational stress; the impacts of stress on performance; self-care; and information about—and practice using—the Big Four techniques of tactical breathing, positive self-talk, mental rehearsal, and goal setting. Booster sessions are available online three months post-training, focusing on the Mental Health Continuum, stigma and discrimination in the workplace, and the Big Four.

#### Appendix A.1.7. Thompson Rivers University Individual Resilience Course

The Individual Resilience Course at Thompson Rivers University is part of a project funded through a Canadian Institutes of Health Research (CIHR) Team Grant focusing on helping public safety employees and their families to build resilience. The course aims to increase individual knowledge of stress and stress mitigation strategies, leading to increased personal resilience. The course consists of 12 modules, covering the topics of: resilience, stress, stress vs. trauma, coping, relaxation, sleep, exercise and physical activity, social support and engagement, other resilience skills, ethics and wellness, discussing mental health, and posttraumatic growth. A revised version of the course is planned for 2026.

### Appendix A.2. Workplace Resilience Courses

#### Appendix A.2.1. Enhancing Psychological Health, Wellness and Resilience

In this brief, self-directed course offered by MyWorkplaceHealth, learners in Enhancing Psychological Health, Wellness and Resilience are presented with three lessons, beginning with an overview of how the COVID-19 pandemic impacted mental health in Canada and reflecting on its impact on one’s own state of mental health. Lesson 2 focuses on recognizing signs of stress, identifying responses, burnout, and identifying strategies for coping. Lesson 3 concludes with information about building resilience and an exercise to develop a personal plan for work–life balance.

#### Appendix A.2.2. Psychological Health and Safety: Building Team Success

Directed toward organizational leaders, Psychological Health and Safety: Building Team Success is a brief, self-directed course that provides education on psychological health and safety. In Module 1, participants are taught about the relationship between mental health and psychological safety, with a focus on mental health stigma, identifying and mitigating situations in the workplace that can harm mental health, responding to mental health crises, protecting confidentiality, and building mental health competency in oneself and among one’s team. Module 2 focuses on the roles and responsibilities of leaders in implementing strategies and plans for psychological health and safety, identifying skills for effective leadership and key competencies to develop. In Module 3, participants identify ways to support the psychological health and safety of their teams and develop a short psychological safety action plan.

#### Appendix A.2.3. Psychological Health and Safety for Employers

Offered by the Canadian Centre for Occupational Health & Safety (CCOHS), the four-module, self-directed Psychological Health and Safety for Employers course is designed for leaders across a variety of occupations. Module 1 introduces the concept of psychological health and safety, why it is important, and barriers. In Module 2, participants learn to assess and identify psychosocial hazards and then apply this knowledge in Module 3 in order to understand how to control such hazards, both in general and by focusing on specific psychosocial factors. Learners then evaluate the effectiveness of these control measures in Module 4 and are reminded of the importance of continuous improvement.

#### Appendix A.2.4. Psychological Health and Safety for Workers

Psychological Health and Safety for Workers is an online, self-directed course offered by the Canadian Centre for Occupational Health & Safety (CCOHS) and is intended for a general audience. Participants are guided through four lessons. The first module introduces participants to the concept of psychological health and safety. Next, participants learn about why psychological health and safety is important, as well as signs of psychologically unsafe workplaces and their effects on health and the workplace, as well as information about the prevalence of mental health concerns in Canada. The subsequent lesson focuses specifically on psychological health and safety at work, including stigma; psychosocial factors; work–life balance; and feeling safe, valued, respected, and supported. Finally, learners are informed about the respective roles that employers and employees have in ensuring a psychologically safe work environment.

#### Appendix A.2.5. Reducing Mental Health Stigma in the Workplace

Reducing Mental Health Stigma in the Workplace is a brief, online, self-directed course designed by the Canadian Centre for Occupational Health & Safety (CCOHS) specifically to raise awareness about mental health stigma. In the first lesson, participants are taught the difference between stereotypes, prejudice, and discrimination. Participants are then informed of ways that mental health stigma is harmful, both for individuals and for the workplace, and are provided with guidance on using person-first rather than identity-first language. Finally, learners are provided with suggestions for how individuals and workplaces can reduce mental health stigma.

#### Appendix A.2.6. Thompson Rivers University Workplace Resilience Course

The Workplace Resilience Course at Thompson Rivers University is part of a project funded through a Canadian Institutes of Health Research (CIHR) Team Grant focusing on helping public safety employees and their families to build resilience. This course aims to increase workplace resilience among public safety employees through psychoeducation. The course consists of six modules, addressing the topics of what workplace resilience is, leadership, civility and inclusion, conflict resolution, bullying and harassment, and ethics and integrity. A revised version of the course is planned for 2026.

### Appendix A.3. Family Resilience Courses

#### Appendix A.3.1. PSPNET Families

PSPNET Families is not a course as such, but rather an online resource that provides a combination of information and skill development tools. Information pages are organized according to five sections: (1) the effects of public safety work and home transitions on the family, (2) the impacts of the job at home, (3) workplace requirements, public expectations, and their effects, (4) public safety personnel (PSP) families’ experience with trauma, and (5) roles and responsibilities in a PSP family. The Strategies and Skill Building section includes numerous tools and activities focusing on families (e.g., childcare, prioritizing family, co-parenting, communication with children, planning for “what-if” scenarios) and couples (e.g., goal setting, discussing trauma, improving sleep, managing conflict, problem solving).

#### Appendix A.3.2. Thompson Rivers University Family Resilience Course

The Family Resilience Course at Thompson Rivers University is part of a project funded through a Canadian Institutes of Health Research (CIHR) Team Grant focusing on helping public safety employees and their families to build resilience. The course is based on Walsh’s resilience framework [47] and is designed to increase family resilience for adults, teens, and children of public safety employees. Across nine lessons, course participants learn about themes such as building confidence, the importance of shared values and traditions, adapting to change, recognizing family contributions, accessing resources, open communication, expressing feelings, and teamwork in decision-making. A revised version of the course is planned for 2026.
